# Glutamine Metabolism Promotes Renal Fibrosis through Regulation of Mitochondrial Energy Generation and Mitochondrial Fission

**DOI:** 10.7150/ijbs.89960

**Published:** 2024-01-12

**Authors:** Yang Cai, Beichen Tian, Yuanjun Deng, Lele Liu, Chunjiang Zhang, Wei Peng, Qian Li, Tianjing Zhang, Min Han, Gang Xu

**Affiliations:** Department of Nephrology, Tongji Hospital, Tongji Medical College, Huazhong University of Science and Technology, 1095 Jiefang Ave., Wuhan 430030, China.

**Keywords:** Renal fibrosis, Glutamine, Fibroblasts, Mitochondria, α-ketoglutaric acid, Mitochondrial fission

## Abstract

Fibroblast activation and proliferation is an essential phase in the progression of renal fibrosis. Despite the recognized significance of glutamine metabolism in cellular growth and proliferation, its precise pathophysiological relevance in renal fibrosis remains uncertain. Therefore, this study aims to investigate the involvement of glutamine metabolism in fibroblast activation and its possible mechanism. Our findings highlight the importance of glutamine metabolism in fibroblast activation and reveal that patients with severe fibrosis exhibit elevated serum glutamine levels and increased expression of kidney glutamine synthetase. Furthermore, the deprivation of glutamine metabolism in vitro and in vivo could inhibit fibroblast activation, thereby ameliorating renal fibrosis. It was also detected that glutamine metabolism is crucial for maintaining mitochondrial function and morphology. These effects may partially depend on the metabolic intermediate α-ketoglutaric acid. Moreover, glutamine deprivation led to upregulated mitochondrial fission in fibroblasts and the activation of the mammalian target of rapamycin / mitochondrial fission process 1 / dynamin-related protein 1 pathway. Thus, these results provide compelling evidence that the modulation of glutamine metabolism initiates the regulation of mitochondrial function, thereby facilitating the progression of renal fibrosis. Consequently, targeting glutamine metabolism emerges as a novel and promising avenue for therapeutic intervention and prevention of renal fibrosis.

## Introduction

Chronic kidney disease (CKD) is a prevalent global public health issue, particularly in light of the aging population and the increasing prevalence of hypertension and diabetes 1. In China, the incidence of CKD has gradually reached approximately 10% 2. Renal fibrosis is a prevalent consequence of chronic and progressive nephropathies, yet its pathogenesis remains elusive 3. Therefore, investigating the pathogenesis of renal fibrosis and identifying effective intervention strategies to delay or potentially reverse the progression of fibrosis represents a challenging and pressing issue in the field of nephrology 4.

Several cell types, such as myofibroblasts and inflammatory cells, participate in the progression of renal fibrosis, which is considered to be the key effector cells 5. Fibroblasts are widely recognized as the primary source of myofibroblasts 6, 7. Myofibroblast differentiation from fibroblast is a critical aspect of the fibrosis process, as it is the primary cause of extracellular matrix production and the development of renal fibrosis 8. Currently, the specific molecular mechanism of fibroblast activation is not well characterized. Therefore, targeting fibroblasts is a promising therapeutic approach for the intervention of renal fibrosis.

A growing number of studies indicate that metabolic reprogramming occurs in various kidney disorders, including acute kidney injury, renal fibrosis, and diabetic kidney disease 9-11. In animal models of unilateral ureteral obstruction (UUO), several metabolic pathways are altered, including increased anaerobic glycolysis, decreased glutamine uptake and oxidation capacity, and a massive reduction in fatty acid uptake and oxidation 12. Therefore, modulation of metabolic processes is expected to become a promising avenue for identifying and managing renal fibrosis. However, the precise participation in metabolism in fibroblast activation remains uncertain.

The kidney, being a vital organ for amino acid metabolism, participates in the unhindered passage of amino acids through the glomerular filtration barrier, followed by their excretion from the body 13. Glutamine, the predominant amino acid in the blood, assumes a crucial function in cellular metabolism 14. Research has shown that glutamine metabolism is increased in tumor cells with vigorous growth and active metabolism and that tumor cell growth can be suppressed by inhibiting glutamine metabolism 15. Metabolomic profiling analyses have unveiled augmented levels of plasma glutamine and glutamate in CKD rat models 16. It is hypothesized that disorders of glutamine metabolism may contribute to the development of renal fibrosis.

Mitochondria, the essential intracellular organelles, are responsible for energy production and a variety of biological processes 17. The significance of mitochondria in kidney injury intervention investigations has been proposed 18. A study has revealed that targeting the serine phosphorylation site 600 of dynamin-related protein 1 (DRP1) could diminish mitochondrial fission events and thereby ameliorate diabetic nephropathy 19. Accumulating evidence also suggests that modifications of cellular metabolism can also impact mitochondrial function. The research findings indicate that inhibition of fatty acid metabolism in renal tubules can reduce adenosine triphosphate (ATP) production, suppress mitochondrial function, and promote cell apoptosis 20. Metabolic regulation of renal fibrosis is thought to be related to the modulation of mitochondrial function 21. However, the mechanisms of glutamine metabolism and mitochondria in renal fibrosis require further investigation.

Consequently, the present study aimed to examine the involvement of glutamine metabolism in fibroblast activation and the possible mechanism related to mitochondrial dysfunction to provide novel perspectives on the management of CKD.

## Methods

Additional detailed methods are included in the Supplemental Methods.

### Animals and unilateral ureteral obstruction

Male C57BL/6 mice (8 weeks old, weighing 20-25g) were obtained from Beijing Weitong Lihua Experimental Animal Company (Beijing, China). To generate fibroblast-specific glutamine synthetase (GLS) deletion mice, GLS^flox/flox^ mice were crossed with transgenic mice carrying S100a4 promoter-driven Cre recombinase, in which Cre recombinase is observed in fibroblasts and a subset of myeloid cells. To induce renal interstitial fibrosis in mice, unilateral ureteral obstruction (UUO) was implemented following established procedures. The Supplementary Methods section contains comprehensive information regarding animal studies.

### Cell culture

The NRK-49F cell lines derived from rat kidney interstitial fibroblasts were acquired from the American Type Culture Collection located in Manassas, USA. Detailed protocols are outlined in the Supplementary Methods.

### Statistical analysis

All data were expressed as means ± standard error (SD). The Supplementary Methods contain comprehensive protocols. Statistical significance was determined by considering probability (p) values below 0.05.

## Results

### Glutamine metabolism is involved in the activation of renal fibroblasts

TGF-β1 (10ng/ml) was used to stimulate rat kidney fibroblasts (NRK-49F) to construct a model of fibroblasts transdifferentiation into myofibroblasts 22-24. We tested the changes in mRNA and protein expression of FN and SMA at different time points. The results detected that mRNA and protein expression of FN and SMA gradually increased, and there was statistical significance at the point of 24h (Figure S1A-B). The evidence shows that the model is successful.

Metabolite changes were monitored by untargeted metabolomics during fibroblast activation. The results showed that fibroblast activation resulted in a significant up-regulation of 65 metabolites and a substantial down-regulation of 30 metabolites, as compared to the control group.

**Figure 1:**
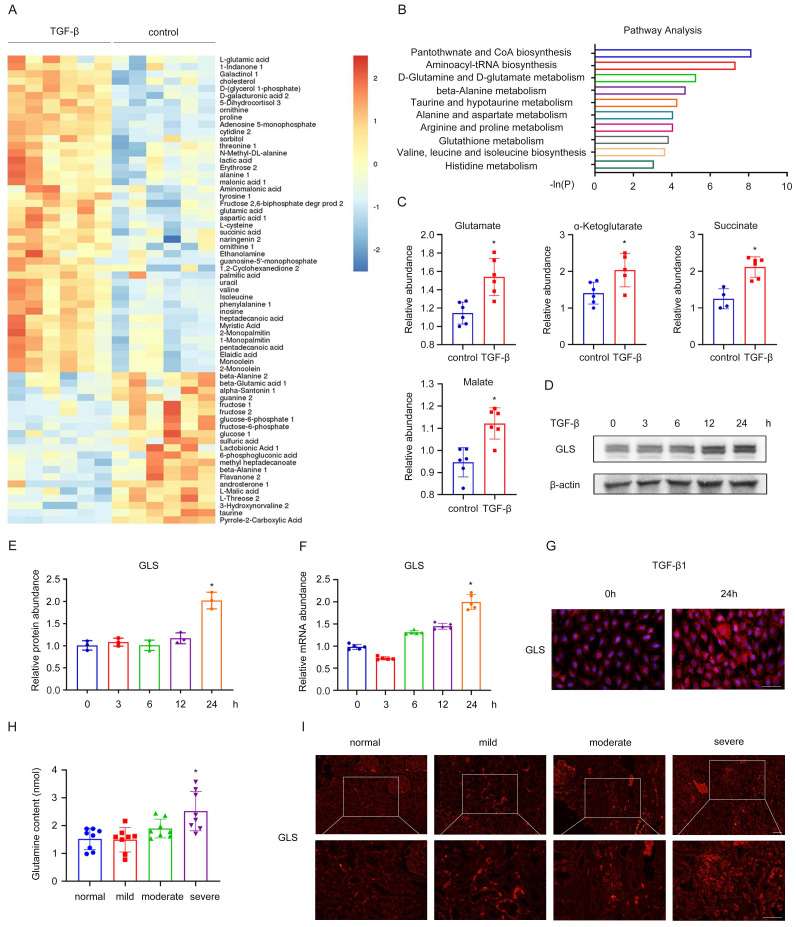
Glutamine catabolism is involved in the activation of renal fibroblasts. (A) We used TGF-β1 to stimulate rat renal fibroblasts (NRK-49F) to construct a model of fibroblasts transdifferentiation into myofibroblasts. Untargeted metabolomics showed changes in metabolites during fibroblast activation. (B) Further metabolic pathway analysis of differential metabolites was assessed. (C) A targeted analysis focusing specifically on glutamine metabolism was performed. (D) and (E) NRK-49F were treated with TGF-β1 for 0h, 3h, 6h, 12h, 24h. The protein level of GLS1 was detected by Western blot analysis. (F) Real-time PCR showed the mRNA level of GLS1. (G) Immunofluorescence staining showed the expression of GLS1. Scale bars are 50 µm. (H) Serum and renal biopsy specimens from IgA nephropathy patients with mild, moderate, and severe fibrosis. The levels of glutamine were examined by ELISA. (I) Immunofluorescence staining detected the expression of GLS1 in renal biopsy specimens. Scale bars are 50 µm. *p < 0.05.

Next, a heatmap of the 95 identified metabolites was then generated and shown in Figure 1A. Through further metabolic pathway analysis of differential metabolites, we found the key pathways most related to the differential metabolites, including pantothenate and CoA biosynthesis, aminoacyl-tRNA biosynthesis, D-Glutamine and D-glutamate metabolism, beta-alanine metabolism, taurine and hypotaurine metabolism (Figure 1B). To confirm our observation, we performed a targeted analysis focusing specifically on glutamine metabolism. An increase of glutamate, α-ketoglutarate, succinate, and malate was detected during fibroblast activation compared with the control group (Figure 1C). To further investigate the impact of glutamine metabolism in the activation of fibroblasts, we used TGF-β1 to stimulate NRK-49F and tested the changes in GLS expression at different time points. It was shown that as the time of TGF-β1 stimulation prolonged, GLS mRNA and protein expression gradually increased by western blot and rt-PCR (Figure 1D-F). Consistent with the above results, cellular immunofluorescence analysis also confirmed significant upregulation of GLS expression after 24 hours of TGF-β1 stimulation (Figure 1G).

A similar result was found in CKD patients. We procured serum and renal biopsy samples from individuals diagnosed with IgA nephropathy, exhibiting diverse levels of renal fibrosis. The experiment results of Masson, HE, and PAS of patients diagnosed with IgA nephropathy, exhibiting diverse levels of renal fibrosis in Figure S1C in supplemental material. Mild, moderate, and severe fibrosis are defined by the area of fibrosis, which is 0-25%, 25-50%, and greater than 50%, respectively. Notably, patients with severe fibrosis displayed elevated serum glutamine levels, as determined by ELISA (Figure 1H). Immunofluorescence results further revealed augmented expression of GLS in the renal interstitium of patients with severe fibrosis (Figure 1I). Thus, glutamine metabolism was involved in the process of fibroblast activation and was significantly increased.

### Renal fibroblast activation is highly dependent on glutamine metabolism

**Figure 2:**
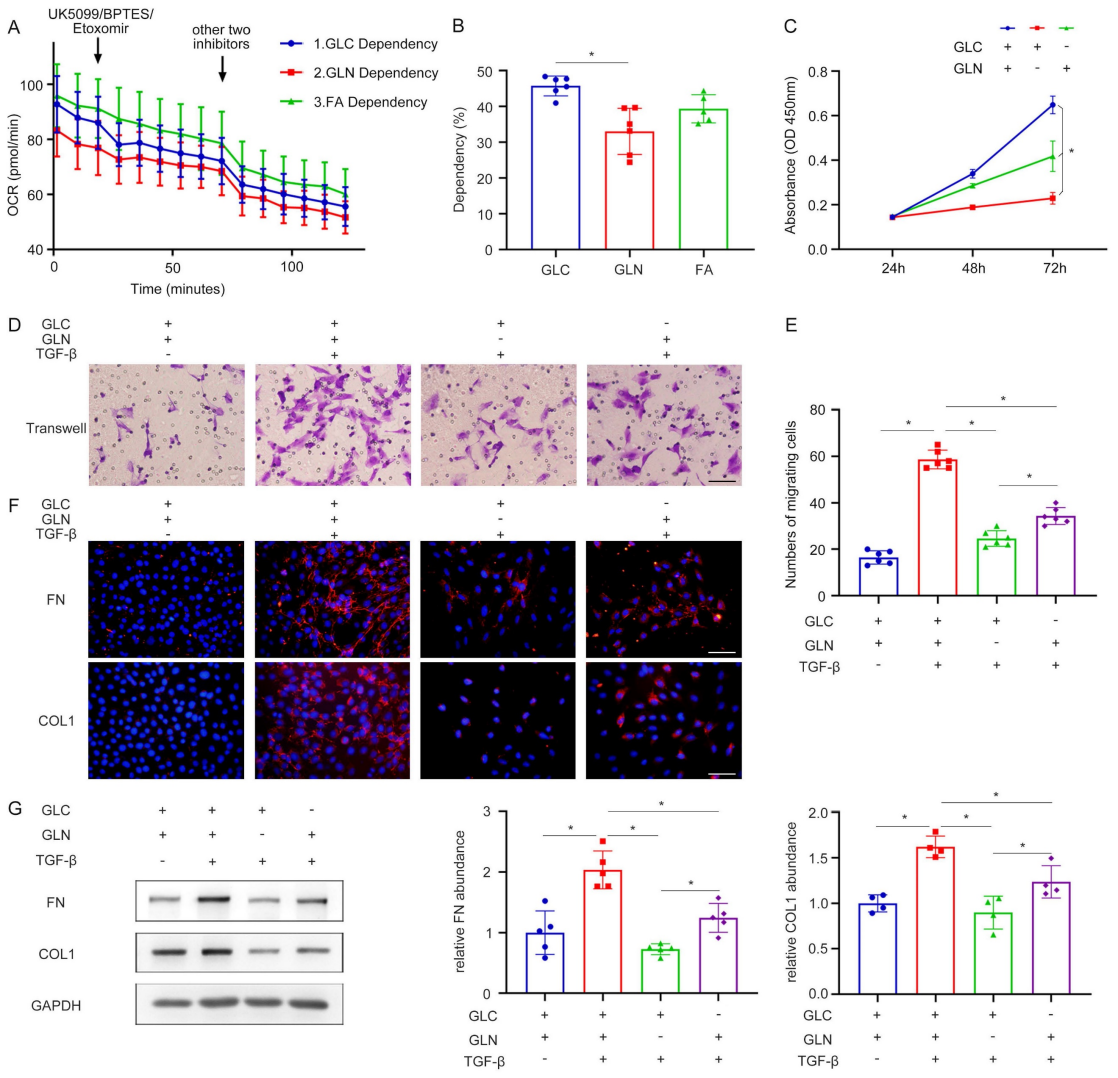
Renal fibroblast activation is highly dependent on glutamine catabolism. (A) and (B) Seahorse energy metabolism instrument detected the dependence of glucose metabolism, fatty acid metabolism, and glutamine metabolism in fibroblast activation. (C) NRK-49F were cultured with glutamine deprivation and glucose deprivation. The proliferation of fibroblast was assessed by the CCK-8 experiment. (D) and (E) Transwell migration assay was performed to calculate the migration of fibroblast. (F) NRK-49F were treated with control, TGF-β1, TGF-β1 with GLN deprivation, and TGF-β1 with GLC deprivation. Immunofluorescence staining showed the expression of FN and COL1. Scale bars are 50 µm. (G) The expression levels of fibrotic markers (FN, COL1) were detected by Western blot analyses. *p < 0.05.

Subsequent experiments were conducted to elucidate the importance of glutamine metabolism in fibroblast activation. The Seahorse Energy Metabolism Instrument was employed to ascertain the predominant metabolic pathway involved in fibroblast activation. The results proved that glucose metabolism was the main metabolic process during fibroblast activation, followed by fatty acid metabolism and then glutamine metabolism (Figure 2A, B). In addition, the roles of glucose metabolism and glutamine metabolism in fibroblast function were evaluated to explain why we chose glutamine metabolism as the subject. Interestingly, several different pieces of evidence were found compared with the results of seahorse energy metabolism. As assessed by the CCK-8 experiment, both glutamine deprivation and glucose deprivation significantly reduced cell viability and the result of glutamine deprivation was more pronounced, demonstrating that fibroblast growth was mainly dependent on glutamine (Figure 2C). Moreover, the transwell migration assay was utilized to observe the migration capacity of fibroblasts. Compared with glucose deprivation, glutamine deprivation inhibited the migration ability of fibroblasts more significantly (Figure 2D, E). It was confirmed by cellular immunofluorescence that in the process of fibroblast activation, both glutamine and glucose deprivation lead to a reduction in fibroblast extracellular matrix secretion, such as fibronectin (FN), collagen I (Col I), and the reduction was more pronounced with glutamine deprivation (Figure 2F). This finding was further supported by the western blot (Figure 2G). These data demonstrated the crucial role of glutamine metabolism in renal fibroblast activation, highlighting their dependence on glutamine rather than glucose for the maintenance of the fibrogenic myofibroblastic phenotype.

### The decrease of glutamine metabolism reduces the activation of kidney fibroblasts

**Figure 3:**
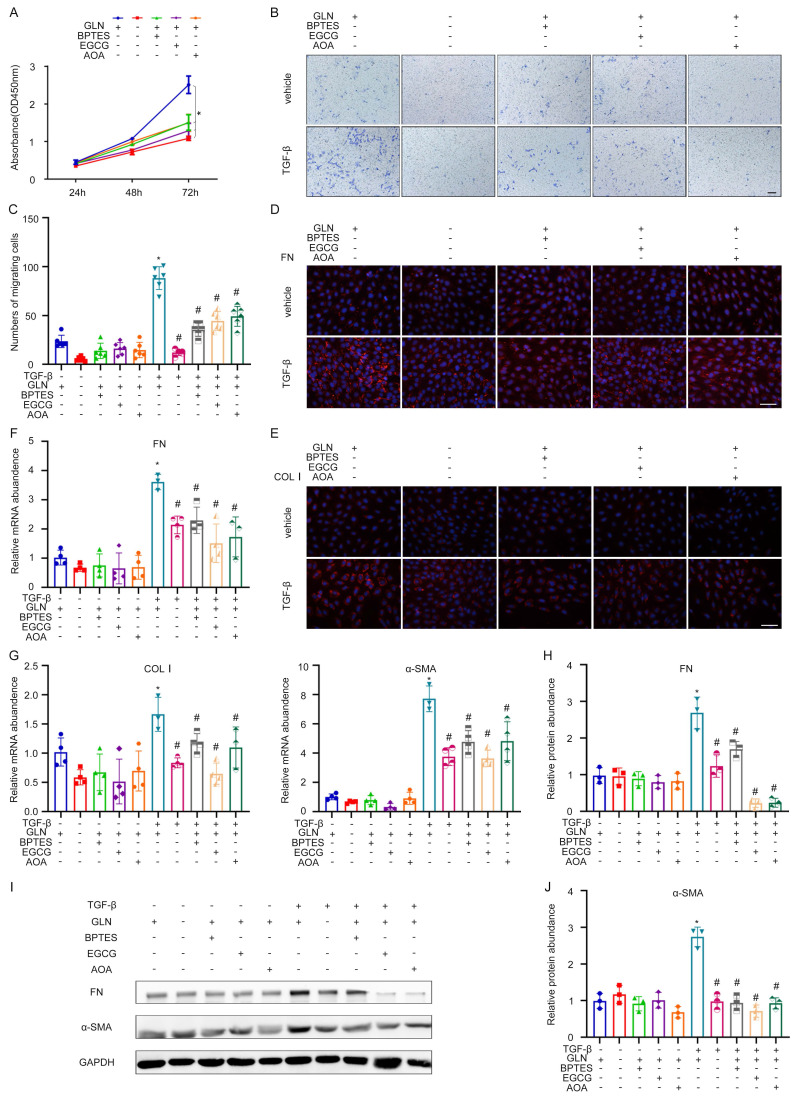
Decrease of glutamine catabolism can reduce the activation of kidney fibroblasts. (A) Glutamine deprivation BPTES, EGCG and AOA can inhibit the glutamine metabolic pathway. The proliferation of fibroblast was assessed by the CCK-8 experiment. (B) and (C) The migration of fibroblast was detected by the Transwell migration assay. Scale bars are 50 µm. (D) and (E) Immunofluorescence staining displayed the expression of FN and COL1. Scale bars are 50 µm. (F) and (G) Real-time PCR showed the mRNA level of fibrotic markers (FN, COL1, and ɑ-SMA). (H) - (J) Western blot showed the protein level of FN and α-SMA. *p < 0.05.

To further explore the effects of GLS on the activation of fibroblasts, we knocked down GLS in NRK-49F cells by using different inhibitors and a specific siRNA. These inhibitors could inhibit the key enzymes in the metabolic pathway of glutamine. Glutamine catabolism occurs in two distinct stages. The initial stage involves the conversion of glutamine to glutamic acid through deamination catalyzed by the enzyme GLS. In the subsequent stage, the conversion of glutamate to α-ketoglutarate is facilitated by the enzymes glutamate dehydrogenase (GDH) and glutamic pyruvic transaminase (GPT). BPTES, EGCG, and AOA are the specific inhibitors of GLS, GDH, and GPT, respectively. The CCK-8 experiment showed that BPTES, EGCG, AOA, and exogenous glutamine deprivation could significantly inhibit the growth of NRK49F (Figure 3A). Through transwell migration assay, it was found that exogenous deprivation of glutamine, BPTES, EGCG, and AOA could significantly inhibit the migration ability of fibroblasts, and the inhibitory effect of exogenous deprivation of glutamine was the most significant, and the cells almost lost their migration ability (Figure 3B, C). Exogenous glutamine deprivation and BPTES, EGCG, and AOA can inhibit the expression of TGF-β1-induced FN, Col I detected by cellular immunofluorescence (Figure 3D, E). The mRNA expression of FN, Col I, and alpha smooth muscle actin (α-SMA) was consistent with the above (Figure 3F, G). The western blot showed the same tendency of protein expression of FN and α-SMA (Figure 3H-J).

Next, we constructed three siRNAs that could specifically silence GLS. After transfection of siRNA, the silencing efficiency of GLS was determined by western blot and rt PCR. It was found that three siRNAs could inhibit the mRNA expression of the GLS gene by about 80 %, and siRNA 02 had the most obvious inhibitory effect at the protein level, so we selected siRNA 02 for subsequent experiments (Figure S2A, B). Western blot demonstrated that silencing GLS could inhibit the expression of FN and α-SMA in the process of fibroblast activation (Figure S2C, D). The immunofluorescence assay of FN was consistent with the above (Figure S2E). Furthermore, the activation of kidney fibroblasts is significantly influenced by glutamine metabolism.

### The maintenance of mitochondrial energy generation is heavily reliant on glutamine metabolism

Since mitochondrial damage has been acknowledged as a primary factor responsible for renal fibrosis 25-27, we tested the mitochondrial function of NRK-49F in the absence of glutamine. The levels of voltage dependent anion channel 1 (VDAC1) and cytochrome c oxidase IV (COX IV) represent the mitochondrial content.

**Figure 4:**
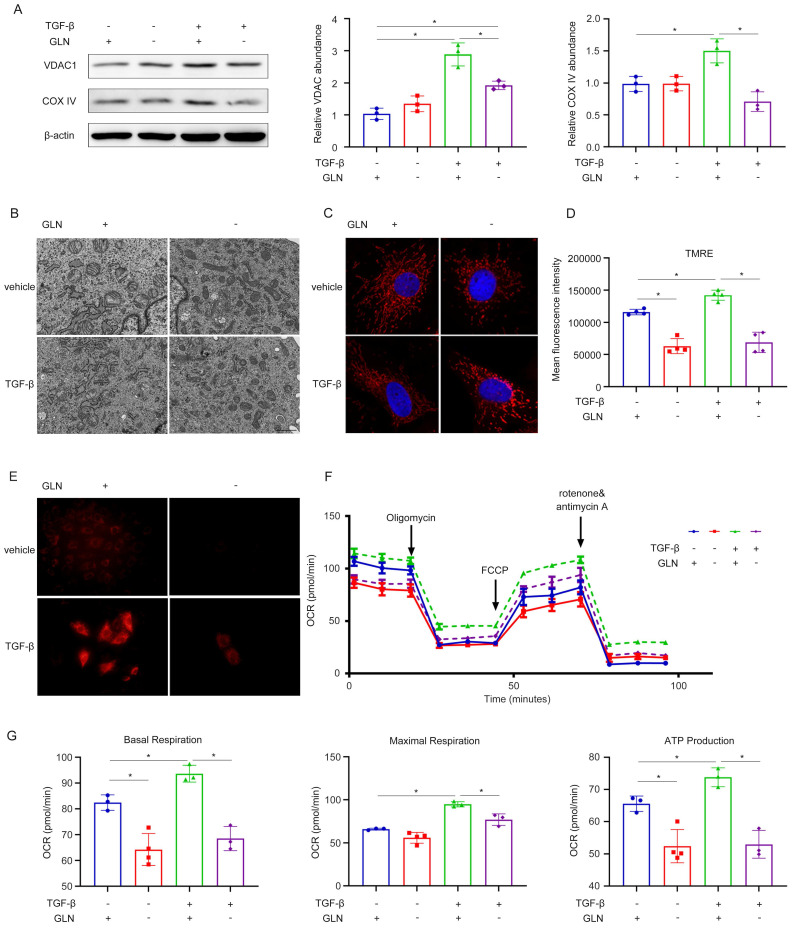
Glutamine catabolism plays an important role in maintaining mitochondrial energy generation. (A) NRK-49F were treated with control, -GLN, TGF-β1, T-GLN. The expression levels of mitochondrial content (VDAC1 and COX IV) were detected by Western blot analyses. (B) Mitochondrial electron microscopy and (C) mitoTraker showed mitochondrial morphology. (D) and (E) TMRE fluorescence determined the strength of mitochondrial membrane potential by fluorescence intensity. (F) and (G) The oxygen consumption rate (OCR) of cells was tested using a seahorse energy metabolism instrument. *p < 0.05.

It was found that the mitochondrial content was significantly increased in the process of fibroblast to myofibroblast transformation induced by TGF-β1, however, this increase could be inhibited by the absence of glutamine (Figure 4A). Mitochondrial morphology was evaluated using mitoTracker. Mitochondria in the control group were filamentous and intertwined into a network, whereas mitochondria in the glutamine deprivation group were fragmented and punctate, whether treated with TGF-β1 or not (Figure 4C). The results of mitochondrial electron microscopy were consistent with the above findings (Figure 4B). TMRE fluorescence labeling can determine the strength of the mitochondrial membrane potential by fluorescence intensity. Flow cytometry was then used to determine TMRE uptake. TGF-β1 stimulation significantly increased TMRE fluorescence, whereas glutamine deprivation could significantly decrease fluorescence (Figure 4D). The fluorescence staining outcomes of TMRE cells corroborated these findings (Figure 4E). This suggested that the mitochondrial membrane potential was significantly increased during the transdifferentiation of fibroblasts into myofibroblasts, which was decreased in the absence of glutamine. Then we investigated the impact of glutamine deprivation on mitochondrial respiratory capacity using a seahorse energy metabolism instrument. Our findings revealed that the oxygen consumption rate (OCR) of cells in the TGF-β1 stimulation group exhibited a significant upregulation compared to the control group, while it was downregulated during glutamine deprivation. Additionally, basal oxygen consumption rate, maximal oxygen consumption rate, and ATP production showed a trend toward the same effect (Figure 4F, G). Taken together, these results confirm that glutamine metabolism regulated the fibroblast activation by maintaining mitochondrial energy generation.

### The activation of renal fibroblasts is significantly influenced by the involvement of α-ketoglutaric acid, an intermediate in glutamine metabolism

**Figure 5:**
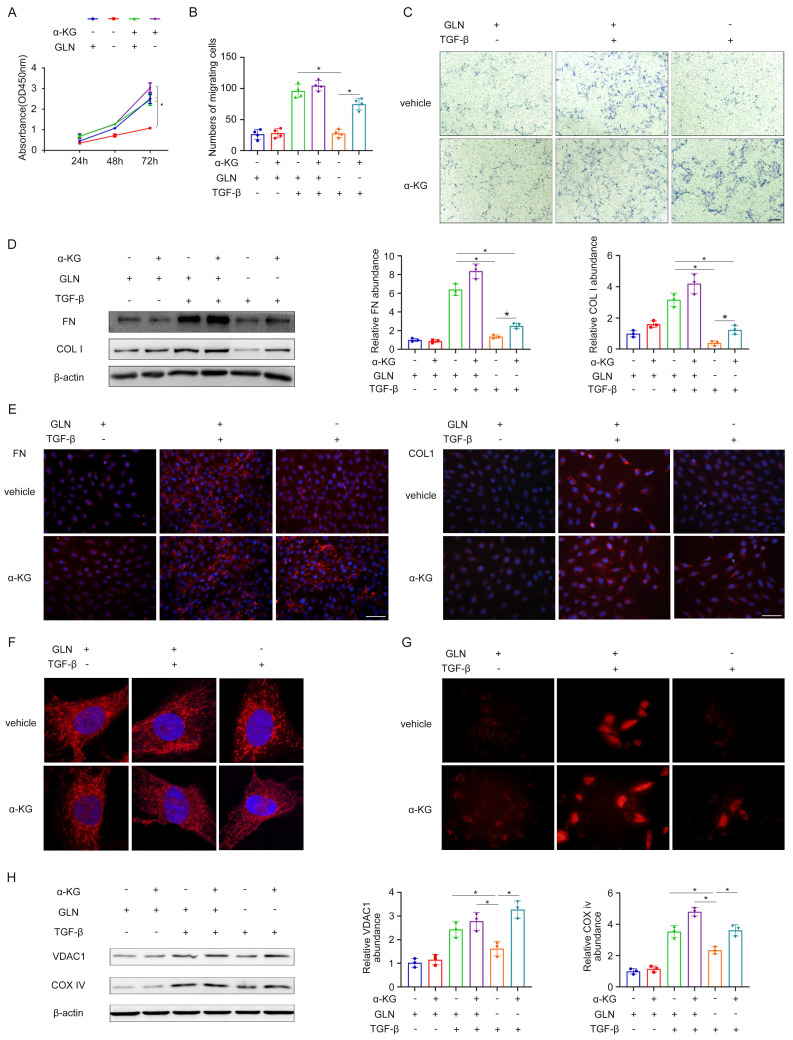
Glutamine catabolism intermediate α-ketoglutaric acid plays an important role in the activation of renal fibroblasts. (A) We supplemented α-ketoglutarate after glutamine deprivation during fibroblast activation. CCK-8 assay evaluated the proliferation of fibroblasts. (B) and (C) The migration of fibroblast was explored by the Transwell migration assay. Scale bars are 50 µm. (D) Western blot showed protein levels of the fibrotic markers (FN and COL1). (E) Immunofluorescence staining in fibroblasts showed the expression levels of FN and COL1. Scale bars are 50 µm. (F) MitoTraker showed mitochondrial morphology. (G) The strength of mitochondrial membrane potential was detected by TMRE fluorescence. (H) The expression levels of mitochondrial content (VDAC1 and COX IV) were displayed by Western blot analyses. *p < 0.05.

α-ketoglutaric acid (α-KG) is a crucial intermediate in the process of glutamine catabolism, which can enter directly into the tricarboxylic acid (TCA) cycle. Given the importance of α-KG in glutamine metabolism, we investigated several properties of fibroblasts and mitochondrial function under glutamine deprivation followed by α-KG supplementation. The results obtained from the CCK-8 experiment and transwell migration assay demonstrated that the absence of glutamine had a substantial inhibitory effect on the proliferation and migration of fibroblasts. However, when α-KG was supplemented following glutamine deprivation, there was a partial restoration observed in both the proliferation rate and migration ability of the fibroblasts (Figure 5A-C). In the process of fibroblast activation, glutamine deprivation led to the decrease of extracellular matrix secretion in fibroblasts, such as FN, COL I, and the addition of α-KG after glutamine deprivation increased the extracellular matrix secretion compared with the simple deprivation of glutamine (Figure 5D, E). This indicated that glutamine deprivation could significantly inhibit TGF-β1-induced fibroblast activation, but supplementation of α-KG after glutamine deprivation could partially restore TGF-β1-induced activation. It was found that by mitoTracker during the process of fibroblast to myofibroblast transformation induced by TGF-β1, mitochondria in the glutamine deprivation group were fragmented and punctate during glutamine deprivation, and then α-KG could reverse the effect to some degree (Figure 5F). TMRE fluorescence confirmed that glutamine deprivation significantly hindered the increase in mitochondrial membrane potential induced by TGF-β1, while α-KG supplementation could partially restore the mitochondrial membrane potential (Figure 5G). By detecting the mitochondrial content of VDAC1 and COX IV, it was found that during the process of fibroblast to myofibroblast transformation induced by TGF-β1, as glutamine deprivation reduced the mitochondrial content of fibroblasts, and then α-KG could reverse the effect to some degree (Figure 5H). Taken together, these data suggest that α-KG could partially reverse the effects caused by glutamine deprivation and glutamine exerted its effect via α-KG during the fibroblast activation.

### Glutamine metabolism regulated the mTOR/MTFP1/DRP1 pathway to modulate the mitochondria fission

**Figure 6:**
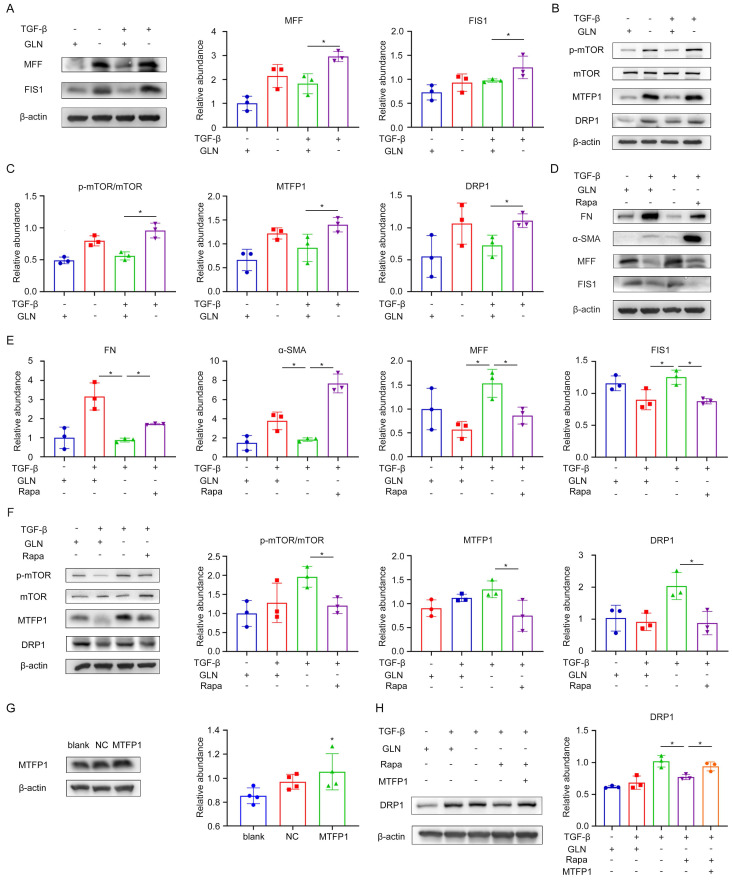
Glutamine metabolism regulated the mTOR/MTFP1/DRP1 pathway to modulate mitochondria fission. (A) Western blot showed protein levels of the mitochondrial fission markers (MFF and FIS1). (B)and (C) Alterations in proteins involved in the mTOR/MTFP1/DRP1 pathway were detected when glutamine was present. (D), (E) and (F) When RAPA was used to especially inhibit the mTOR activity, the levels of fibrotic markers (FN and COL1), MFF/FIS1, and mTOR/MTFP1/DRP1 pathway were examined by western blot. (G) MTFP1 overexpression plasmid was transfected into fibroblast to determine the relationship between MTFP1 and DRP1. Western blot showed the transfection efficiency of MTFP1 and the accordingly change of DRP1. (H) DRP1 expression was detected under MTFP1 overexpression by Western blot. *p < 0.05.

Based on the data described above, we found that mitochondria were fragmented in the absence of glutamine during fibroblast activation. Mitochondrial fission factor (MFF) and mitochondrial fission protein 1 (FIS1) play critical roles in mitochondrial fission 28. The levels of MFF and FIS1 were efficiently increased during glutamine deprivation by Western blot (Figure 6A). The mammalian target of rapamycin (mTOR) is a nutrient sensor that senses glutamine abundance and can be activated by glutamine metabolism 29. Mitochondrial fission process 1 (MTFP1) is a mitochondrial inner membrane protein whose overexpression promotes mitochondrial fragmentation 30. DRP1 is a GTPase involved in mitochondrial fission and is the primary implementer of fission 31.

To examine the involvement of the mTOR/MTFP1/DRP1 pathway in the process of fibroblast activation, protein expression along this pathway was assessed using western blot analysis (Figure 6B, C). The results revealed that mTOR activity was enhanced and the protein levels of MTFP1 and DRP1 were elevated when glutamine was absent during fibroblast activation. Furthermore, we treated the fibroblasts with rapamycin (RAPA), a specific mTOR inhibitor, to elucidate the role of mTOR in this pathway. Rapa treatment reversed the reduction of extracellular matrix secretion and the increased expression of mitochondrial fission protein caused by glutamine deprivation in fibroblasts, as indicated by western blot analysis (Figure 6D, E). As for the changes in the pathway, the protein expression of MTFP1 and DRP1 was decreased by mTOR inhibition (Figure 6F). To further investigate whether mTOR regulates DRP1 expression by modulating MTFP1, we upregulated MTFP1 expression through a MTFP1 plasmid while using RAPA and observed the levels of DRP1 protein. It was found that the reduction of DRP1 by RAPA was reversed by the upregulation of MTFP1 (Figure 6G, H). Collectively, these results revealed that glutamine metabolism regulated the mitochondria fission by modulating the mTOR/MTFP1/DRP1 pathway during the fibroblast activation.

### Inhibition of glutamine metabolism alleviates renal fibrosis in the UUO model

**Figure 7:**
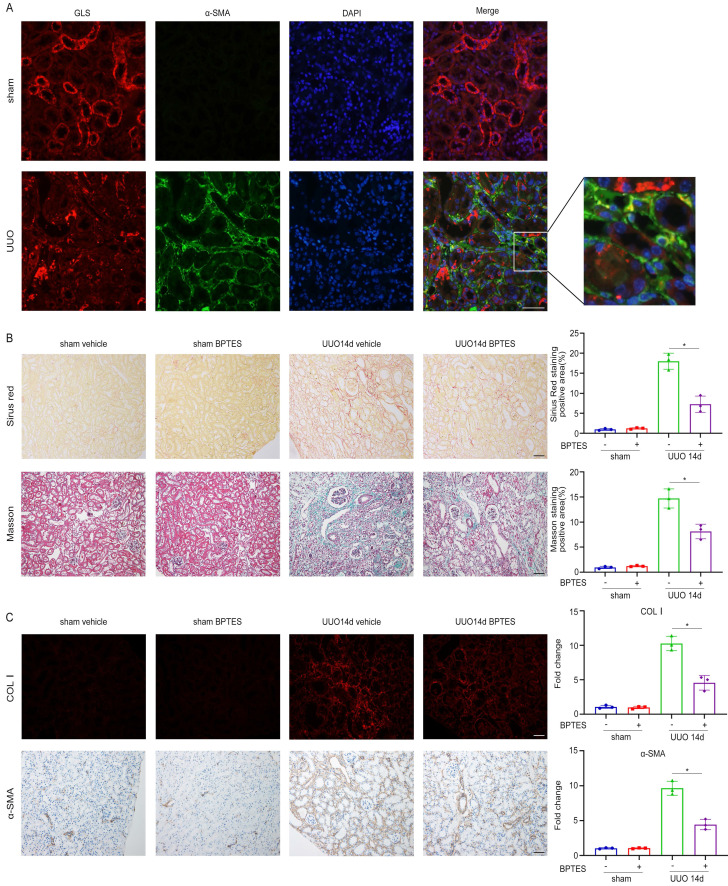
Inhibition of glutamine catabolism can inhibit UUO-induced renal fibrosis. UUO mice were treated with BEPTES. On day 14, mice were sacrificed, and the kidneys were collected. (A) Immunofluorescence staining of GLS1(red) and α-SMA (green) in the UUO kidneys on day 14. Scale bars are 50 µm. (B) Collagen staining was showed by Masson and Sirius red on day 14 after UUO injury. Scale bars are 50 µm. (C) Immunofluorescence and immunohistochemistry staining detected the expression of COL1 and α-SMA. Scale bars are 50 µm. *p < 0.05.

In our in vitro experiments, we demonstrated that inhibition of glutamine metabolism could suppress TGF-β1-induced fibroblast activation by impairing mitochondrial function. To test whether glutamine metabolism exhibited a similar function in vivo, we utilized the UUO model to induce renal fibrosis over a period of 14 days. Through immunofluorescent staining of GLS1 and α-SMA, it was found that there was fluorescence co-staining of partial GLS1 and α-SMA in the kidney of UUO, indicating that the expression of GLS1, the key enzyme of glutamine metabolism, was increased in some myofibroblasts (Figure 7A). To assess the impact of glutamine metabolism on renal fibrosis, we injected BPTES, a specific inhibitor of GLS, intraperitoneally on the 3rd, 5th, and 7th days after the establishment of the UUO model. Renal interstitial fibrosis was markedly attenuated in the BPTES group, as evidenced by Sirius Red and Masson staining in renal sections when compared to the solvent control group (Figure 7B). Immunofluorescence analysis further revealed a notable decrease in the expression of Col I in the BPTES group compared to the solvent control group. Additionally, the expression of α-SMA was diminished in the BPTES group compared to the solvent control group (Figure 7C).

**Figure 8:**
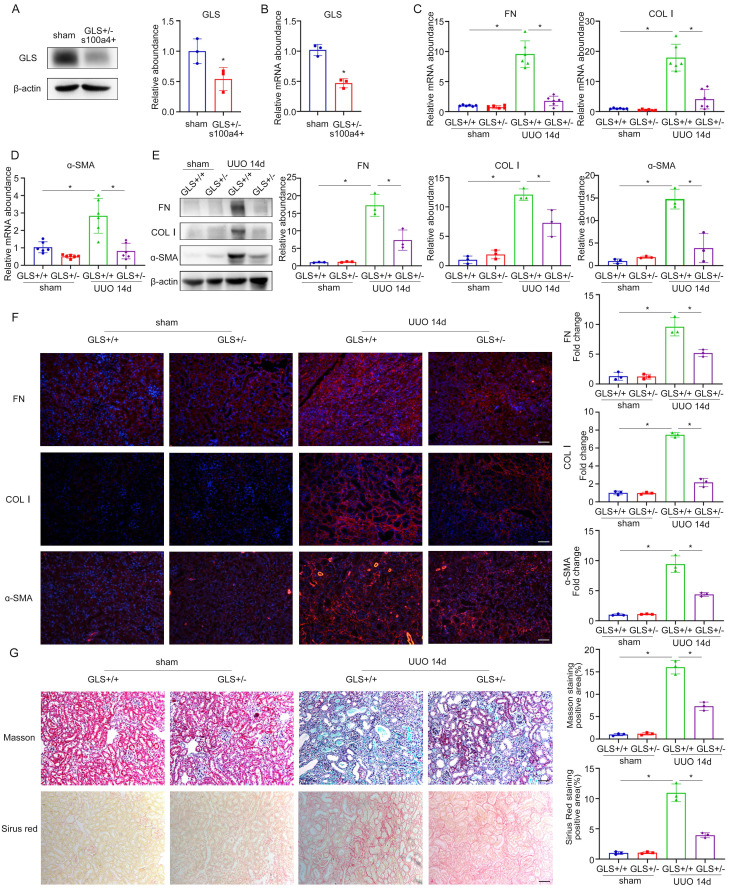
Specific knockout of GLS1 in fibroblasts can inhibit UUO-induced renal fibrosis. (A) and (B) GLS^flox/flox^ mice were crossed to transgenic mice carrying S100a4 promoter-driven Cre recombinase to generate fibroblast-specific GLS deletion mice. The knockout efficiency of GLS1 was detected by western blot and real-time PCR. (C) and (D) Real-time PCR showed the mRNA level of fibrotic markers (FN, COL1, and ɑ-SMA). (E) The protein levels of FN, COL1, and ɑ-SMA were evaluated by Western blot analyses. (F) Immunofluorescence staining displayed the expression of FN, COL1, and ɑ-SMA. Scale bars are 50 µm. (G) Masson and Sirius's Red staining indicated collagen deposition. Scale bars are 50 µm. *p < 0.05.

To elucidate the involvement of glutamine metabolism in fibroblasts, we developed a murine model of GLS deletion in renal fibroblasts. GLS^flox/flox^ mice were crossed with transgenic mice carrying S100a4 promoter-driven Cre recombinase, in which Cre recombinase is observed in fibroblasts and a subset of myeloid cells. We used heterozygotes to construct the UUO model for 14 days, because the complete knockout of GLS may affect the survival of homozygotes. The knockout efficiency of GLS in heterozygotes was approximately 50% (Figure 8A, B). Sirius Red and Masson staining indicated that compared with the wildtype group, renal interstitial fibrosis was significantly attenuated under the GLS knockdown (Figure 8G). To further confirm whether renal fibrosis induced by UUO was reduced by GLS knockdown, we detected the expression of fibrotic markers (FN, COL Ⅰ, and ɑ-SMA). The outcomes of western blot and rt-PCR demonstrated that kidneys of the GLS+/- group secreted less extracellular matrix than the wildtype group (Figure 8C-E). Immunofluorescent staining results further confirmed the above findings (Figure 8F). These data provided strong evidence to conclude that inhibition of glutamine metabolism could prevent UUO-induced renal fibrosis by modulating the activation of fibroblast.

Then we explored the involvement of the mTOR/MTFP1/DRP1 pathway in the UUO model. The outcomes of western blot demonstrated that mTOR activity was enhanced and the protein levels of MTFP1 and DRP1 were elevated in the kidneys of conditional GLS knockout mice group than the wildtype group, which are consistent with the cell results (Figure S3A). Immunofluorescence assay of MTFP1 and DRP1 corroborated the above findings (Figure S3B). These results revealed the modulation of the mTOR/MTFP1/DRP1 pathway in the UUO model of conditional GLS knockout mice.

## Discussion

Renal fibrosis is widely recognized as the prevailing terminal phase of progressive renal disease, leading to impaired renal function 32. Given the lack of effective therapeutic interventions for renal fibrosis, it is crucial to discover novel targets for addressing this condition. Numerous investigations have unveiled the association between multiple abnormal metabolisms and the development of renal fibrosis, resulting in the accumulation of certain metabolites and affecting cellular function and disease progression 33-35. Despite these understandings, the specific role and mechanism of glutamine metabolism in renal fibrosis remain unclear. In the present investigation, we elucidated the importance of glutamine metabolism in fibroblast activation and found that intervention of glutamine metabolism could inhibit fibroblast activation and thereby ameliorate renal fibrosis. Furthermore, it has been determined that the maintenance of mitochondrial function is reliant upon glutamine metabolism. These effects may depend on the metabolic intermediate α-KG. Additionally, glutamine metabolism regulated the mTOR/MTFP1/DRP1 pathway to modulate mitochondrial fission during fibroblast activation. Collectively, these findings substantiate the notion that glutamine metabolism promotes renal fibrosis through the regulation of mitochondrial function, thus offering novel perspectives and potential therapeutic targets for the prevention and treatment of renal fibrosis.

Previous studies have demonstrated that glutamine metabolism was enhanced when quiescent hepatic stellate cells differentiated into myofibroblast. Inhibition of glutamine metabolism could suppress the activation of hepatic stellate cells, thereby attenuating liver fibrosis 36. The current study also detected a similar phenomenon, as non-target metabolomics showed that during the process of renal fibroblast activation, glutamine metabolic processes were significantly upregulated. Moreover, glutamine metabolism was enhanced in both the renal fibrosis model of UUO mice and IgA patients with severe renal fibrosis. The study revealed that fibroblast activation in renal fibrosis is primarily characterized by a shift in metabolic activity from oxidative phosphorylation to aerobic glycolysis, commonly referred to as the Warburg effect 37. Furthermore, it was observed that the inhibition of aerobic glycolysis in renal fibroblasts can effectively mitigate renal fibrosis 38. Thus, we compared the effects of glucose metabolism and glutamine metabolism during fibroblast activation. Interestingly, several different pieces of evidence were found compared with the results of seahorse energy metabolism. The findings demonstrated that while glucose metabolism played a major role in fibroblast activation, compared with glucose deprivation, glutamine deprivation led to more reduction of the proliferative and migration capability and fewer secretion of the extracellular matrix. These data demonstrated the crucial role of glutamine metabolism in renal fibroblast activation, highlighting the more dependence on glutamine rather than glucose for the maintenance of the fibrogenic myofibroblastic phenotype. Therefore, we chose glutamine metabolism as the object of study. Therefore, it is proposed that fibroblast proliferation and acquisition of a myofibrogenic phenotype are more dependent on glutamine metabolism than glucose metabolism.

Given the importance of glutamine metabolism, the involvement of glutamine metabolism in fibroblast activation was investigated. In vitro inhibition of glutamine metabolism suppressed the proliferation and migration of fibroblasts, thereby diminishing the secretion of extracellular matrix. The UUO mice exhibited a similar effect, as demonstrated by intraperitoneal injection of the glutamine-specific inhibitor BPTES, which effectively suppressed glutamine metabolism in vivo, thereby inhibiting fibroblast activation, reducing the deposition of extracellular matrix and attenuating renal fibrosis. Moreover, specific knockout of GLS in fibroblasts led to a significant improvement in renal fibrosis. This phenomenon has also been documented in liver and lung diseases, where the suppression of glutamine metabolism has been shown to inhibit fibroblast activation and attenuate fibrosis in these organs 39-41. The metabolite α-KG plays a pivotal role in glutamine metabolism, as demonstrated by Deepti Singh who observed a significant improvement in cell growth rate upon α-KG supplementation during fibroblast culture. Our present study provides additional insight into the impact of glutamine deprivation on fibroblast proliferation, migration, and extracellular matrix secretion, which can be partially restored by α-KG supplementation following glutamine deprivation. Notably, these findings suggest a potential mechanism in which glutamine metabolism provides nitrogen for the synthesis of some non-essential amino acids through deamination 42. These results provide compelling evidence that glutamine metabolism is indispensable for the proliferation and activation of fibroblasts, which is partially dependent on α-ketoglutaric acid generated from glutamine.

The mitochondria, a pliable reticulum organelle, serve a crucial function in the maintenance of various cellular processes that are implicated in renal fibrosis 43. Myofibroblasts, characterized by strong contractility, can synthesize and secrete numerous extracellular matrix 44. The maintenance of these functions requires a large amount of ATP synthesized by mitochondrial oxidative phosphorylation 45. The augmentation of glutamine metabolism could enhance the activity of the TCA cycle, promote ATP generation, and synthesize intermediate metabolites for nucleic acids, amino acids, and lipids 46. Therefore, we hypothesize that regulation of mitochondrial function could serve as a possible mechanism through which glutamine metabolism modulates fibroblast activation. The results evinced a significant enhancement in mitochondrial function during TGF-β1 induced fibroblast activation, possibly attributable to the enormous augment in oxidative phosphorylation and glycolysis during fibroblast activation, as confirmed by Bernard et al 47. Glutamine deprivation has the potential to reduce the augmented mitochondrial function, which can be partially restored by the addition of α-ketoglutarate. Our findings parallel the previous literature observing similar results in hepatic stellate cells 36. In summary, it suggests that glutamine metabolism participated in fibroblast activation by preserving mitochondrial function.

The relationship between mitochondrial morphology and function is a critical aspect of cellular physiology. Mitochondria are interwoven into reticulation and the dynamic equilibrium between mitochondrial fusion and fission is preserved under physiological conditions 18. This balance can be broken under external stimuli or pressure, resulting in the fragmentation of mitochondria into smaller fragments, called mitochondrial fission, and ultimately leading to mitochondrial dysfunction. 48. Bernard et al. reported an increase in podocyte mitochondrial fragmentation in diabetic nephropathy, which could lead to mitochondrial dysfunction and reduce cellular ATP production 49. The current investigation depicts a significant increase in mitochondrial fragments upon suppression of glutamine metabolism, suggesting that glutamine metabolism is a crucial factor in preserving the typical morphology of mitochondria.

Furthermore, our study provides evidence for a potential association between the mTOR/MTFP1/DRP1 pathway and fibroblast activation under conditions of glutamine deprivation. Interestingly, our results indicated that mTOR activity was enhanced when glutamine was absent, which is contrary to the conventional pathway. Multiple studies believe that glutamine can activate mTORC1 through the Rag-independent pathway and that mTORC1 activity is inhibited following glutamine deprivation 50, 51. And some studies are consistent with the findings in this study 52-54. Zhao et al. discovered that the induction of Parkinson's disease cell model in vitro leads to the inhibition of the PI3K/Akt/mTOR signaling pathway by glutamine. Consequently, this inhibition enhances the antioxidant capacity of nerve cells and protects against oxidative stress. Additionally, glutamine deprivation resulted in the upregulation of MTFP1 and DRP1, which are involved in the mitochondrial division, suggesting that glutamine depletion may lead to an increase in mitochondrial fission and mTOR/MTFP1/DRP1 pathway involved in the process of fibroblast activation during glutamine deprivation. Rapa treatment reversed the reduction of extracellular matrix secretion and the increased expression of mitochondrial fission protein caused by glutamine deprivation in fibroblasts. This demonstrated glutamine metabolism can regulate mitochondrial morphology via mTOR. Further investigation revealed that mTOR regulates both MTFP1 and DRP1 as downstream targets, and the reduction of DRP1 by RAPA was reversed by the upregulation of MTFP1. These findings align with prior research that has established a connection between the mTOR signaling pathway, mitochondrial dynamics, and cell survival, and the mTOR/4E-BP pathway adjusts mitochondrial fission by regulating the transcriptional translation of MTFP1 55. Simula et al. discovered that programmed death receptor-1 (PD‐1) signaling attenuates mitochondrial fragmentation of T cells by the downregulation of DRP1 phosphorylation on Ser616 through mTOR pathways 56. These unraveled that glutamine metabolism regulated mitochondria fission by modulating the mTOR/MTFP1/DRP1 pathway during the fibroblast activation.

One limitation of this study is that it only explored one pathway in the mechanism of glutamine metabolism in the regulation of fibroblast activation, while glutamine metabolism may play a role through various other mechanisms besides mitochondrial dysfunction. Another limitation is the use of a single animal model, which may introduce bias due to differences between the animal model and human disease.

In conclusion, glutamine metabolism played a crucial role in fibroblast activation. Glutamine deprivation suppressed the ability of fibroblast and mitochondrial function, which could be partially restored by α-KG. Glutamine metabolism modulated mitochondrial fission through the mTOR/MTFP1/DRP1 pathway, thereby regulating fibroblast activation.

## Supplementary Material

Supplementary figures and methods.

## Figures and Tables

**Figure 9:**
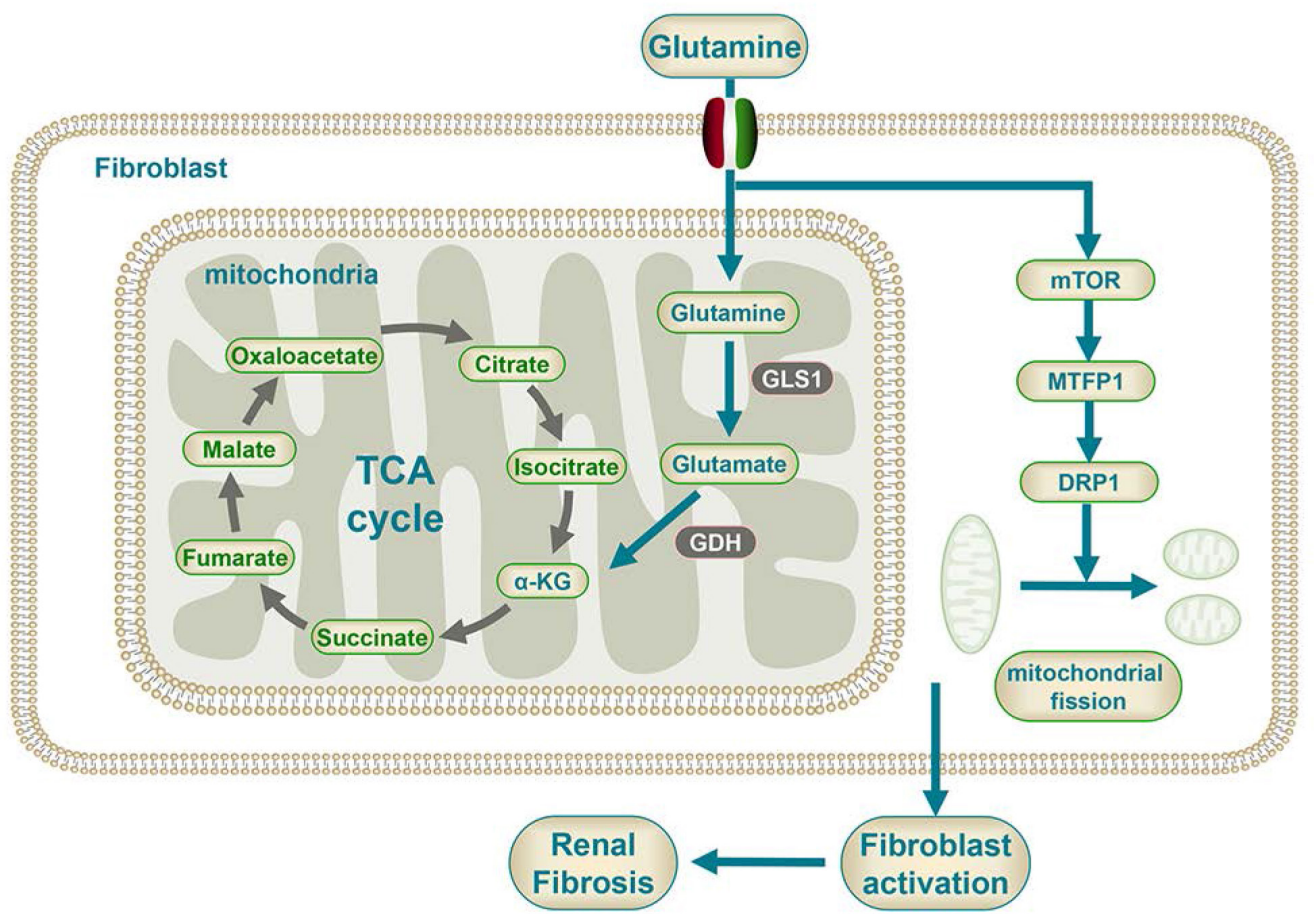
Mechanism diagram of glutamine metabolism participating in the fibroblast activation.
