# Supplementary material for: Glutamine Metabolism Promotes Renal Fibrosis through Regulation of Mitochondrial Energy Generation and Mitochondrial Fission
Source: Int J Biol Sci. 2024 Jan 12;20(3):987–1003. doi: 10.7150/ijbs.89960 (PMC10797689; doi:10.7150/ijbs.89960)
Supplement: Supplementary file 1 — Supplementary figures and methods. [file ijbsv20p0987s1.pdf]

Supplementary Figures

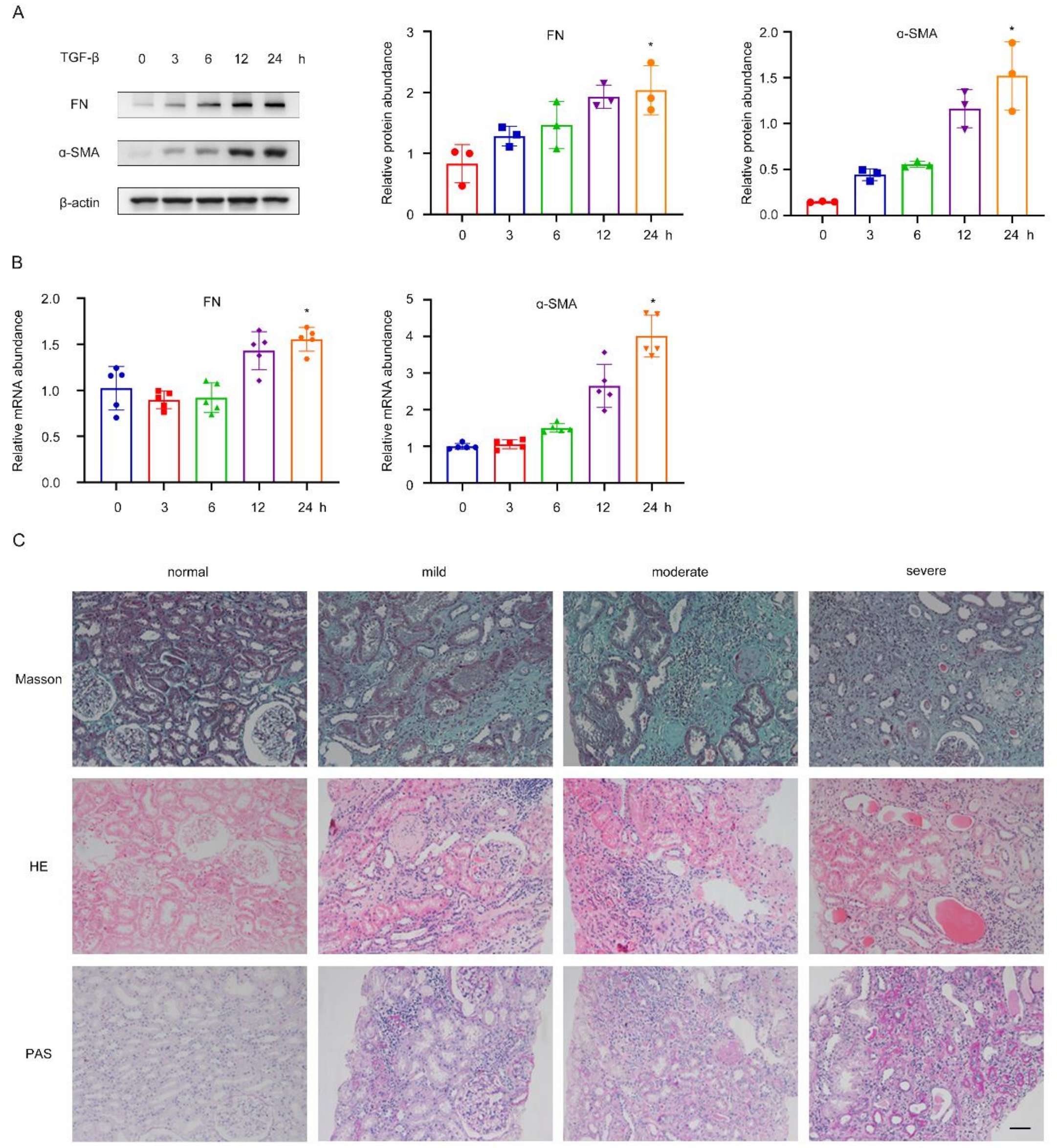

Figure S1

(A) NRK-49F were treated with TGF-β1 for 0h, 3h, 6h, 12h, 24h. The protein level of FN and α-SMA was detected by Western blot analysis.

(B) Real-time PCR showed the mRNA level of FN and α-SMA.

(C) Masson, HE and PAS indicated collagen deposition. Scale bars are 50 μm.

*$p < 0.05$

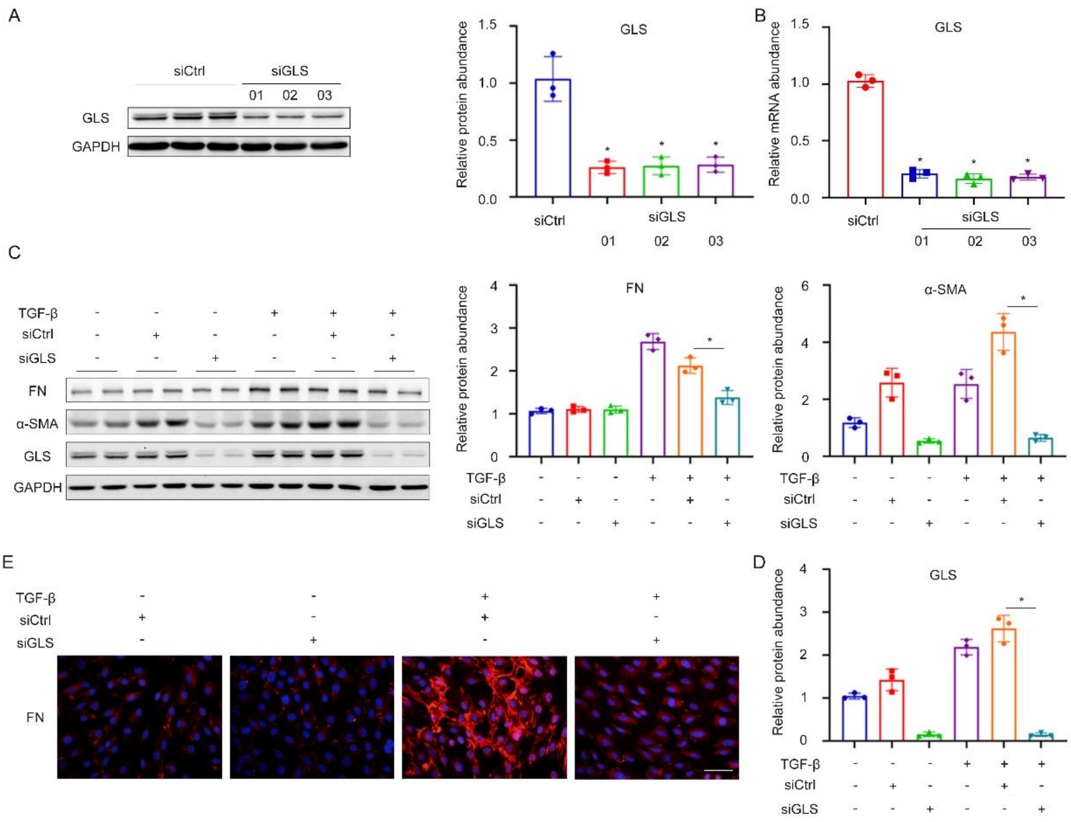

Figure S2

(A)The relative protein level of GLS1 at 24 h after siRNA transfection.

(B) Real-time PCR showed the mRNA level of GLS1 at 24 h after siRNA transfection.

(C) and (D) The protein level of FN, α-SMA, and GLS was evaluated by Western blot analyses.

(E) Immunofluorescence staining displayed the expression of FN. Scale bars are 50 μm.

*$p < 0.05$

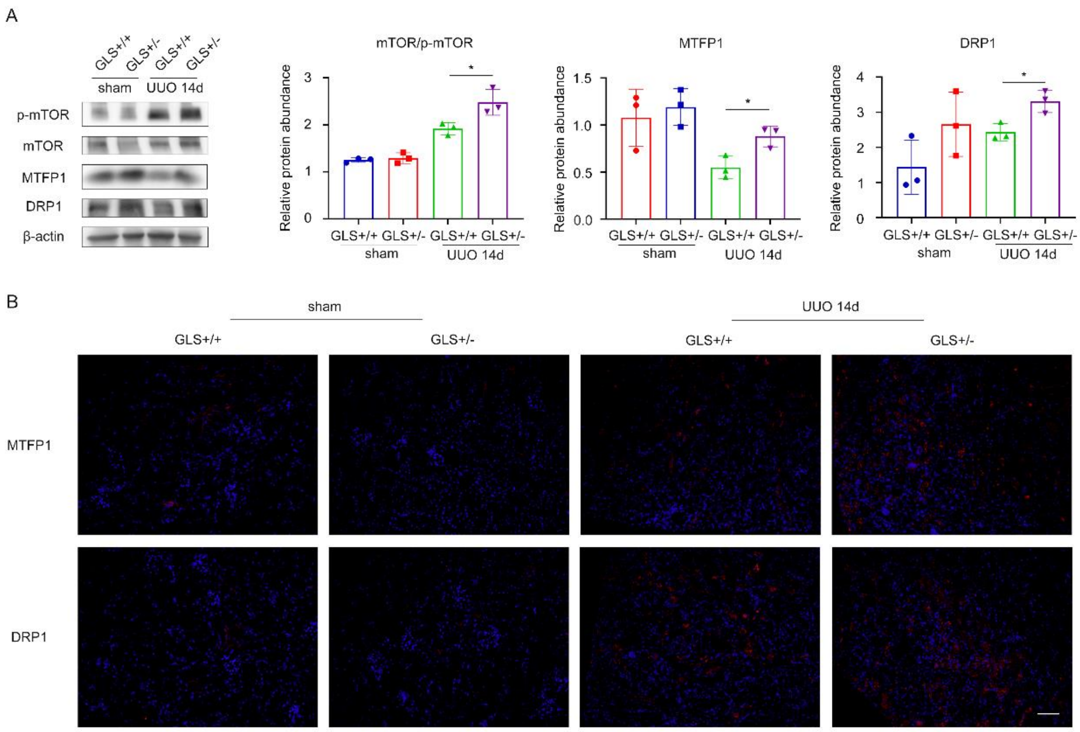

Figure S3

(A) Alterations in proteins involved in mTOR/MTFP1/DRP1 pathway.

(B) Immunofluorescence staining displayed the expression of MTFP1, DRP1. Scale bars are 50 μm.

$*p < 0.05$

Supplementary Methods

Animals and unilateral ureteral obstruction

Unilateral ureteral obstruction (UUO)-induced mouse renal interstitial fibrosis was constructed according to the reported method. Firstly, animals were anesthetized with 1% sodium pentobarbital by i.p. injection. The unilateral dorsal incision was made, then the left ureter was exposed and ligated. All mice were euthanized on the 14th day after surgery and the kidneys were fixed in 4% paraformaldehyde for further analysis simultaneously. All procedures of animails were approved by the Ethics Committee of

Tongji Medical College, Huazhong University of Science and Technology, and conducted according to the Guide for the Care and Use of Laboratory Animals (National Institutes of Health (NIH), Bethesda, MD, USA).

Male C57BL/6 mice (8 weeks old, weighing 200–25g) were obtained from Beijing Weitong Lihua Experimental Animal Company (Beijing, China). Animals were bred and maintained in the Tongji Medical College of Huazhong University of Science and Technology. The mice were randomly and equally allocated into four distinct groups: the sham group, the sham + BPTES group, the UUO group, and the UUO + BPTES group. Each group consisted of three mice. The allocation was performed by a technician who was not involved in the research, using a table of random numbers. The degree of fibrosis was evaluated through the assessment of fibrotic tissue using histological staining and the quantitative analysis of fibrosis-related markers.

To generate fibroblast-specific GLS deletion mice, $GLS^{flox/flox}$ mice were crossed with transgenic mice carrying S100a4 promoter-driven Cre recombinase, in which Cre recombinase is expressed in fibroblasts and some myeloid cells. Identification of the mouse genotypes was carried out by genomic PCR analysis of tail DNA using the following primers: GLS genotyping, 5'-GGCCTGCTTAATGTTTCCTG-3', 5'-GGCATATCCCTGAGTTCGAG-3'; Cre transgene, 5'-TCGATGCAACGAGTGATGAG-3', 5'-TCCATGAGTGAACGAACCTG-3', 5'-CAAATGTTGCTTGTCTGGTG-3', 5'-GTCAGTCGAGTGCACAGTTT-3'. 12 mice were divided into four distinct groups: the GLS +/+ group, the GLS +/- group, the UUO + GLS +/+ group, and the UUO + GLS +/- group. Each group consisted of three mice.

The extent of fibrosis was assessed by histopathological staining and examination of fibrosis-specific markers.

Histological analysis.

Kidneys were fixed in 4% paraformaldehyde and then embedded in paraffin. The 4 μm renal sections were stained to assess the renal tubular damage. Damaged tubules were defined based on tubular dilation, brush border loss, necrosis, and cast formation. Masson and Sirus Red staining were performed to determine the degree of renal interstitial fibrosis. Measurement of the fibrotic area was quantified with ImageJ software (NIH).

Immunohistochemistry and immunofluorescence staining

Paraffinized renal sections were deparaffinized in xylene and hydrated using consecutive ethanol washes of 100%, 95%, 75%, and 50%, and antigen retrieval was performed using citrate solution. The endogenous peroxidase was blocked with 3% H2O2 for 15 minutes, non-specific proteins were blocked with 10% goat serum for 30min. Then, the sections were incubated with primary antibodies of GLS1 (Proteintch, USA), Fibronectin (Proteintch, USA), Collagen I (Proteintch, USA), α-SMA (Abcam, USA) at 4°C overnight. For immunohistochemistry, the slides were then incubated with HRP-conjugated secondary antibody at room temperature for 30 min and visualized with diaminobenzidine substrate. The nucleus was counterstained with hematoxylin, and the slides were visualized under a light microscope. The sections were exposed to

Cy3-labeled secondary antibodies for immunofluorescence and were observed with a fluorescence microscope after staining the nucleus with DAPI.

Human samples

Serum and renal biopsy samples were obtained from patients diagnosed with IgA nephropathy, displaying varying degrees of renal fibrosis. Samples were provided by the Department of Pathology, Tongji Hospital of Huazhong University of Science and Technology. The studies involving human kidney sections and serum were conducted with the informed consent of the patients and were approved by the Institutional Ethics Committee at Tongji Hospital of Huazhong University of Science and Technology. Renal pathologists, who were unaware of the patients' clinical data, assessed interstitial inflammation and interstitial fibrosis (mild, moderate, or severe) in accordance with established pathology guidelines.

Cell culture

The rat kidney interstitial fibroblast cell lines (NRK-49F) were purchased from American Type Culture Collection (Manassas, USA). The cells were routinely cultured in Dulbecco's Modified Eagle's Medium (Gibco, USA) supplemented with 10% fetal bovine serum (Gibco, USA), 100 IU/ml penicillin, and 100 μg/ml streptomycin at 37°C under 5% CO2. Cells that reached approximately 60% confluence were used for in vitro experiments. For the transforming growth factor (TGF)-β1-induced fibrosis model, cells were incubated in a serum-free culture medium for 24 h before exposure to 10

ng/ml recombinant human TGF-β1 (Peprotech, USA) for 24 h.

Cell transfection

For small interfering RNA (siRNA) experiments, GLS siRNA or scrambled siRNA were designed and synthesized. Cells were further transfected with siGLS according to experimental design. Infection efficiency was estimated by western blot. For transfection, cells were seeded in 6-well plates, grown overnight until 60–70% confluent, and then transfected with 50 nM (final concentration) GLS siRNA. Six hours after transfection, the cells were treated with 10 ng/ml TGF-β1.

Untargeted metabolomics

Non-targeted global metabolomic profiles were generated through Appiled Protein Technology (Shanghai) by employing ultra-performance liquid chromatography coupled with high resolution/accurate mass spectrometer (UPLC-MS/MS). To detect a comprehensive range of metabolites, four platforms were utilized: 1) UPLC-MS/MS with positive ionization, 2) UPLC-MS/MS with negative ionization, 3) UPLC-MS/MS polar platform with negative ionization, and 4) gas chromatography-MS. Metabolites were identified based on their m/z retention time and by comparing them to library entities of purified known standards.

Metabolites were analyzed using electrospray ionization (ESI) in auto MS/MS positive scan mode, with a range of 20-1,300 m/z. The ESI source utilized dry nitrogen gas at a flow rate of 10 L/min and a drying temperature of 220˚C. The ESI capillary voltage

was set at 4,500 V with a nebulizer pressure of 2.2 bar. For MS2 acquisition, the collision energy was set at 20 eV and the end Plate Offset at 500 V. Separation of metabolites was achieved using a Hamilton® Intensity Solo 2 C18 column (100 mm × 2.1 mm × 1.8 μm), with sodium formate used as the calibrant for external calibration. The gradient program employed a flow rate of 0.250 ml/min, utilizing a composition of 99A:1.0B from 0.00-2.00 min, transitioning to a composition of 99A:1.0B to 1.0A:99B from 2.00-17.00 min, maintaining a composition of 1.0A:99B from 17.00-20.00 min, and then transitioning back to a composition of 1.0A:99B to 99A:1.0B from 20.00-20.10 min. Subsequently, the flow rate was adjusted to 0.350 ml/min, maintaining a composition of 99A:1.0B from 20.10-28.50 min. Finally, the flow rate returned to 0.250 ml/min, with a composition of 99A:1.0B from 28.50-30 min, resulting in a total run time of 30 min. The maximum pressure reached during the process was 14993 pounds per square inch (PSI). The autosampler temperature was set at 8˚C, while the column oven temperature was maintained at 35˚C. A total volume of 10 μl was injected into the QTOF MS.

Seahorse Energy Metabolism Instrument

The XFe-96 Extracellular Flux Analyzer (Seahorse Bioscience, Agilent, Santa Clara, CA, USA) was utilized to conduct the measurements. NRK-49F cells were seeded into Seahorse tissue culture plates (Seahorse XF96 V3 PS Cell Culture Microplates #101085–004) at a density of 2000 cells per well. Prior to loading into the XF-96 apparatus, the cells were incubated in a CO2-free incubator to achieve equilibration.

After 48 hours, the cells were subjected to two washes and then incubated in a basal Krebs-Ringer bicarbonate HEPES (KRBH) buffer. This buffer consisted of 2.5 mM glucose, 140 mM NaCl, 3.6 mM KCl, 0.5 mM NaH2PO4, 0.5 mM MgSO4, 1.5 mM CaCl2, 10 mM HEPES, and 5 mM NaHCO3, with a pH of 7.4. The incubation took place for a duration of 30 minutes at a temperature of 37°C within the Seahorse instrument. The respiration rates were then measured every 6 minutes at 37°C using the following protocol: 3 minutes of mixing, followed by 3 minutes of measuring oxygen consumption.

Cell proliferation assay

The CCK-8 kit was utilized to assess cell viability, with cells being cultured under the same conditions as previously described. A total of 1×10^4 cells per well were seeded in 96-well plates. Following the treatment, each well received 10 µL of CCK-8 reagent and underwent an incubation period of 1 to 4 hours at 37 °C. The microplate reader was employed to measure the absorbance value at 450 nm, thereby evaluating cell viability. Additionally, blank wells containing only culture media and CCK, as well as control wells consisting of untreated cells, culture media, and CCK, were also subjected to analysis

Transwell Migration Assay

The migration of NRK-49F was assessed using an 8-µm pore size transwell system (Costar). The upper chamber was inoculated with 1 × 104 NRK-49F cells in FBS-free

medium, which were cultured under various conditions for 24 hours. The lower chamber was filled with medium containing 10% FBS to provide a chemotaxis force. Following a 24-hour incubation period, the cells in the upper chamber were fixed with 4% paraformaldehyde for 30 minutes and then stained with 0.1% crystal violet. After removing the non-migratory cells from the inner side of the upper chamber, the migratory cells adhering to the lower surface of the membrane were observed.

Transmission Electron Microscopy

NRK-49F cells cultured on plates were collected and prepared for assessing mitochondrial morphology using a transmission electron microscope provided by the Electron Microscope Center of Renmin Hospital of Wuhan University.

MitoTracker and TRME

To measure mitochondria and mitochondrial membrane potential, MitoTracker deep red (9082P, Cell Signaling Technology) and Tetramethylrhodamine, Ethyl Ester, Perchlorate (TMRE, T669, Thermo Fisher Scientific) staining were employed. Prior to fluorescence activation, NRK-49F cells were incubated in MitoTracker (100 nM) or TMRE (200 nM) at 37°C in the absence of light for 30 minutes.

RNA Extraction and quantitative real-time PCR

Total RNA was extracted from renal tissues or cultured cells by Trizol reagent (TaKaRa, Japan), and 2 μg of RNA was reverse transcribed into cDNA by the PrimeScript RT

Master Mix (Takara, Japan) following the manufacturer's instructions. Quantitative real-time PCR (qRT-PCR) was performed using SYBR Master-Mix (TaKaRa, Japan) under the LightCycler 480 System (Roche, Pleasanton, CA, USA). Through the $2^{-\Delta\Delta Ct}$ method differences in relative expression levels of the target gene were calculated. The specific primers for rat were designed as follows: β-actin, 5′-TCACCCACACTGTGCCCCATCTACGA-3′ (forward) and 5′-CAGCGGAACCGCTCATTGCCAATGG-3′ (reverse); Fibronectin, 5′-GCTTCAAGCTGGGTGTACGA-3′ (forward) and 5′-AAGTTGGTTGGGGGAGACAG-3′ (reverse); Collagen I, 5′-AACCCCAAGGAGAAGAAGCA-3′ (forward) and 5′-AGCGTGCTGTAGGTGAATCG-3′ (reverse); α-SMA, 5′-GTGATCACCATCGGGAATGA-3′ (forward) and 5′-CAGCAATGCCTGGGTACATG-3′ (reverse).

Protein Extraction and Western blotting

Cells and kidney tissues were lysed in RIPA lysis buffer containing protease inhibitor cocktail and phenylmethylsulfonyl fluoride for 30 minutes on ice. Protein concentration was quantified via a BCA assay kit (Promoter, Wuhan, China) following the manufacturer's instructions. Equal amounts of protein were resolved using 10% sodium dodecyl sulfate-polyacrylamide gel electrophoresis (SDS-PAGE) and then blotted onto polyvinylidene difluoride (PVDF) membranes. Following blockage of non-specific antigen, the membranes were incubated overnight at 4°C with the following primary

antibodies: GAPDH (Abcam, USA), β-actin (Abcam, USA), GLS1 (Proteintech, USA), Fibronectin (Proteintech, USA), Collagen I (Proteintech, USA), α-SMA (Abcam, USA), VDAC1 (Cell Signaling Technology, USA), COX IV (Cell Signaling Technology, USA). After washing with TBST and incubated with HRP-conjugated anti-IgG (Servicebio, Wuhan, China), the target protein bands were detected using an enhanced chemiluminescence (ECL) system (Servicebio, Wuhan, China). Quantity One software (BioRad, Hercules, CA, USA) was used to analyze the density of bands.

Statistical analysis

All data were expressed as means ± standard error (SD). Comparisons of two groups were determined by Student’s t-test. Differences between multiple groups were evaluated using one-way ANOVA followed by Tukey's multiple comparisons test. Probability (P) values less than 0.05 were considered statistically significant. All graphs were analyzed using GraphPad (La Jolla, CA, USA) Prism Software 7.0.
